# Easy-to-use background score for routine prostate MRI

**DOI:** 10.1186/s13244-025-02200-5

**Published:** 2026-01-26

**Authors:** Carolin Reischauer, Fabio Porões, Julian Vidal, Hugo Najberg, Nassim Tawanaie Pour Sedehi, Mariem Ben Salah, Johannes M. Froehlich, Harriet C. Thoeny

**Affiliations:** 1 Department of Medicine, University of Fribourg, Fribourg, Switzerland; 2 Department of Radiology, Cantonal Hospital Fribourg, Fribourg, Switzerland; 3 Neurology Unit, Faculty of Science and Medicine, University of Fribourg, Fribourg, Switzerland

**Keywords:** Prostatic neoplasm, Multiparametric magnetic resonance imaging, Diagnosis, Background signal intensity changes, Scoring system

## Abstract

**Objectives:**

To propose an easy-to-use binary scoring system for background signal intensity changes in prostate MRI that may affect diagnostic image interpretation and to evaluate its impact on cancer detection.

**Materials and methods:**

This retrospective single-center study included 200 patients. Four readers independently assigned background scores of A or B according to the proposed scoring system and assessed the presence or absence of cancer. Light’s kappa was used to evaluate inter-reader agreement on the score and on the presence of clinically significant prostate cancer in dependence of the score. Sensitivity and specificity in detecting clinically significant cancer were assessed relative to histology as the gold standard.

**Results:**

Due to suboptimal image quality according to the PI-QUAL score, 45 patients were secondarily excluded. Inter-reader agreement on the score was substantial (kappa = 0.62, 95% CI = 0.54–0.71). Inter-reader agreement on the presence of cancer was higher for a background score A (kappa = 0.49, 95% CI = 0.38–0.61) than B (kappa = 0.34, 95% CI = 0.20–0.51). Sensitivity in detecting cancer was high regardless of the background score (86.61% and 89.42% for scores A and B), while specificity decreased markedly in readers with little experience (53.47% and 43.75% for scores A and B), potentially increasing false positives.

**Conclusion:**

After further validation, the easy-to-use binary background score could enable routine evaluation of normal changes in the peripheral zone, identifying cases with increased false-positive risk among inexperienced readers.

**Critical relevance statement:**

The easy-to-use binary background score for daily clinical routine allows the communication of potential diagnostic uncertainties in mpMRI image interpretation of the prostate that arise due to normal changes in the peripheral zone, especially for less experienced readers.

**Key Points:**

- An easy-to-use binary scoring system for addressing background signal intensity changes in the prostate is proposed for MRI interpretation.
- Inter-reader agreement of the score was substantial, and agreement between readers regarding the presence or absence of cancer was higher for a background score of A than B.
- The background score could be used to communicate a potential diagnostic uncertainty related to the normal change in the peripheral zone, particularly for less experienced readers.

**Graphical Abstract:**

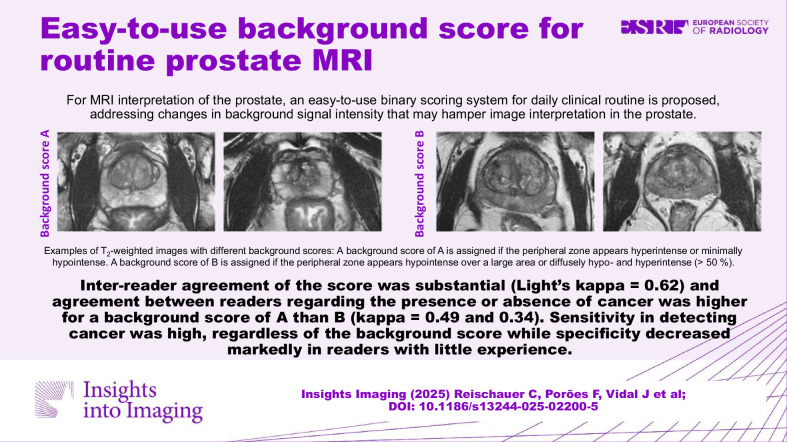

## Introduction

The adoption of multi-parametric magnetic resonance imaging (mpMRI) for the diagnosis of clinically significant prostate cancer has substantially improved patient care in recent years. The introduction of the PI-RADS (Prostate Imaging-Reporting and Data System) guidelines and their subsequent updates [1–3] have made a major contribution to this, leading to a higher degree of standardization and consistency in the image acquisition and interpretation of mpMRI. To assess diagnostic image quality, the Prostate Imaging Quality (PI-QUAL) score has been developed [4] and has recently been updated (version 2) [5]. It is a composite measure that combines a set of objective criteria reflecting the technical image parameters proposed in the PI-RADS guidelines, such as the field of view, with a set of subjective criteria, such as the absence of artifacts in the image. Regardless, low signal intensity on T_2_-weighted imaging in benign situations, for instance, due to prostatitis, benign prostatic hyperplasia, scarring, and post-biopsy hemorrhage, can mimic tumors or obscure lesions on T_2_-weighted imaging, especially in the peripheral zone [6–8]. In addition, signal intensity in the peripheral zone varies with age, with younger men exhibiting lower signal intensity on T_2_-weighted imaging [9]. For this reason, 4-point [9] and 5-point rating scales [10] have been proposed to assess prostate signal intensity homogeneity on prostate MRI examinations but transition into routine clinical practice has not taken place due to the ever-increasing workload. In the present study, a simplified binary background score that is easier to implement in routine clinical practice is proposed. The agreement of the score between readers with varying experience is evaluated and its influence on cancer detection is assessed.

## Materials and methods

### Patient population and MRI acquisition

This retrospective, single-center study was approved by the Cantonal Ethical Committee of Vaud with BASEC number 2020-01859. A consecutive group of 200 patients undergoing mpMRI of the prostate at our institution between 22nd of October 2018 and 31st of May 2019 was included. All patients underwent mpMRI of the prostate at 3 T (Discovery MR750 3.0 T, GE Healthcare) in the supine position in agreement with PI-RADS version 2.1 guidelines [1–3], including T_1_-weighted imaging (Dixon), T_2_-weighted imaging, diffusion-weighted imaging (DWI), and dynamic contrast-enhanced MRI. To mitigate image artifacts, all patients rectally self-administered a laxative cleansing enema (Freka-Clyss® 133 mL, Fresenius Kabi) 15 min prior to the exam and were given scopolamine butylbromide (Buscopan®, 20 mg, Sanofi-Aventis) intravenously immediately prior to the exam. All examinations were performed without an endorectal coil for signal reception.

### Easy-to-use scoring system

**Fig. 1:**
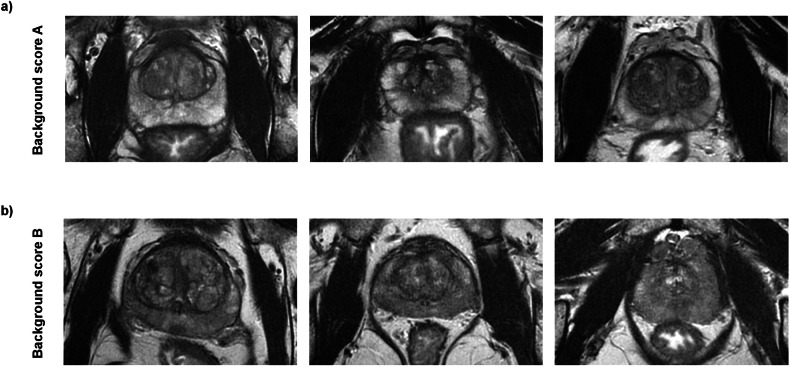
Examples of T_2_-weighted images with different background scores: **a** A background score of A is assigned if the peripheral zone appears hyperintense or minimally hypointense. **b** A background score of B is assigned if the peripheral zone appears hypointense over a large area or diffusely hypo- and hyperintense

Previously proposed background scores consisted of 4 or 5-point rating scales, respectively [9, 10]. To enable applicability in routine clinical practice despite the ever-increasing workload, we propose the use of a binary scoring system in this paper. A score of A is assigned if the peripheral zone appears homogeneously hyperintense or minimally hypointense in less than 50% of the peripheral zone on T_2_-weighted imaging. Contrary, a score of B is assigned if it appears hypointense over a large area of > 50% of the peripheral zone. Examples for different ratings are depicted in Fig. 1.

### Image analysis and standard of reference

Four readers with 4, 4, 6, and 18 years of experience (readers 1–4), who were blinded to all clinical data, biopsy history, and histopathological outcomes, independently reviewed all MRIs retrospectively. To rule out insufficient technical image quality as a confounding factor when analyzing the background score, the three readers with 4, 4, and 6 years of experience (readers 1–3) first assessed the PI-QUAL score (version 1) of each examination [4]. An MRI examination was secondarily excluded from further analysis if any of the three readers did not assign the maximum PI-QUAL score to the respective examination. All four readers then assigned a background score on T_2_-weighted imaging according to the proposed scoring system, excluding areas clearly suspicious for prostate cancer. Each reader additionally assessed the presence or absence of cancer in the peripheral zone, considering all sequences of the mpMRI examination of the respective patient.

The presence of prostate cancer was assessed relative to histology as the standard of reference, either by biopsy or radical prostatectomy, with prostate cancer being considered clinically significant if any of the following criteria were met: Gleason score ≥ 7/ISUP grade group ≥ 2; volume > 0.5 mL and/or extraprostatic extension [3]. Tumor size and extraprostatic extension were estimated on radical prostatectomy when available. The absence of clinically significant cancer in a patient was based on histology or follow-up of the patient as follows: (1) biopsy-proven Gleason score < 6 or Gleason 6 / ISUP grade group 1 not meeting the size/extension criteria for a significant cancer; (2) PI-RADS 1–2 exams with clinical and biological follow-up not requiring subsequent MRI or biopsy. If a follow-up MRI was performed and was classified as PI-RADS ≤ 2, the patient was still included; (3) PI-RADS 3 exams with follow-up MRI at a minimum of 12 months showing no progression.

### Statistical analysis

All statistical analyses were conducted using the computing environment R (R Foundation for Statistical Computing, Vienna, Austria, version 4.3.1). A *p*-value of < 0.05 was used as significance threshold. In all directional (i.e., one-sided) tests, the background score A was expected to return better results than B, as a poorer image quality (i.e., score B) cannot be interpreted as a better performance than a better image quality (i.e., score A). Inter-reader agreement on the background score across all readers was evaluated with Light’s kappa (defined as the average of all possible pairs of readers’ Cohen’s kappa) computed alongside its bootstrapped 95% confidence interval (95% CIs) with 10,000 iterations using the R package *psy* (psy: Various Procedures Used in Psychometrics, Bruno Falissard, R package version 1.2). Inter-reader agreements on the background score between two readers were evaluated with pairwise Cohen’s kappa with 95% CIs using the R package *DescTools* (DescTools: Tools for Descriptive Statistics, Andri Signorell, 2023, R package version 0.99.49). Kappa values were interpreted as follows: ≤ 0.2 = none to slight, 0.21–0.40 = fair, 0.41–0.60 = moderate, 0.61–0.80 = substantial, and 0.81–1.00 = almost perfect agreement [11].

In addition to inter-reader agreement on the background score, the agreement between the readers on the presence of prostate cancer in the peripheral zone, in dependence of the background score, was calculated. In this analysis, the background score determined by the expert reader with 18 years of experience (reader 4) served as the reference standard. Differences in agreement on the presence of cancer in dependence of the background score were assessed using a one-sided *t*-test.

For each reader, the sensitivity and specificity in detecting clinically significant cancer in the peripheral zone were calculated in dependence of the background score, and 95% bootstrap CIs were computed. Again, the background score determined by the expert reader served as the reference standard. Diagnostic performance in dependence of the background score was compared using directional tests of two proportions [12].

As mentioned in the introduction, signal intensity in the peripheral zone on T_2_-weighted imaging varies with age [9], so differences in variables such as age between the two patient groups could influence the results. For this reason, the differences between the two cohorts with different background scores in terms of patient age, prostate volume and prostate-specific antigen (PSA) levels were analyzed using Cohen’s d. If differences in any of these variables, defined as Cohen’s d ≥ 0.5, had been found between the groups, patients furthest from their group’s average would have been removed until the threshold value was reached to ensure a balance between conditions, removing the potential influence of these confounding variables [13, 14].

## Results

### Patient population

A total of 200 patients were primarily included. To rule out technical image quality as a confounding factor, 45 patients were secondarily excluded from the analysis due to suboptimal technical image quality according to the PI-QUAL score. The PI-QUAL scores stratified by background scores for each of the three readers are provided in Supplementary Material 1. Thus, all included patients had the maximum PI-QUAL score, as determined independently by three readers.

**Fig. 2:**
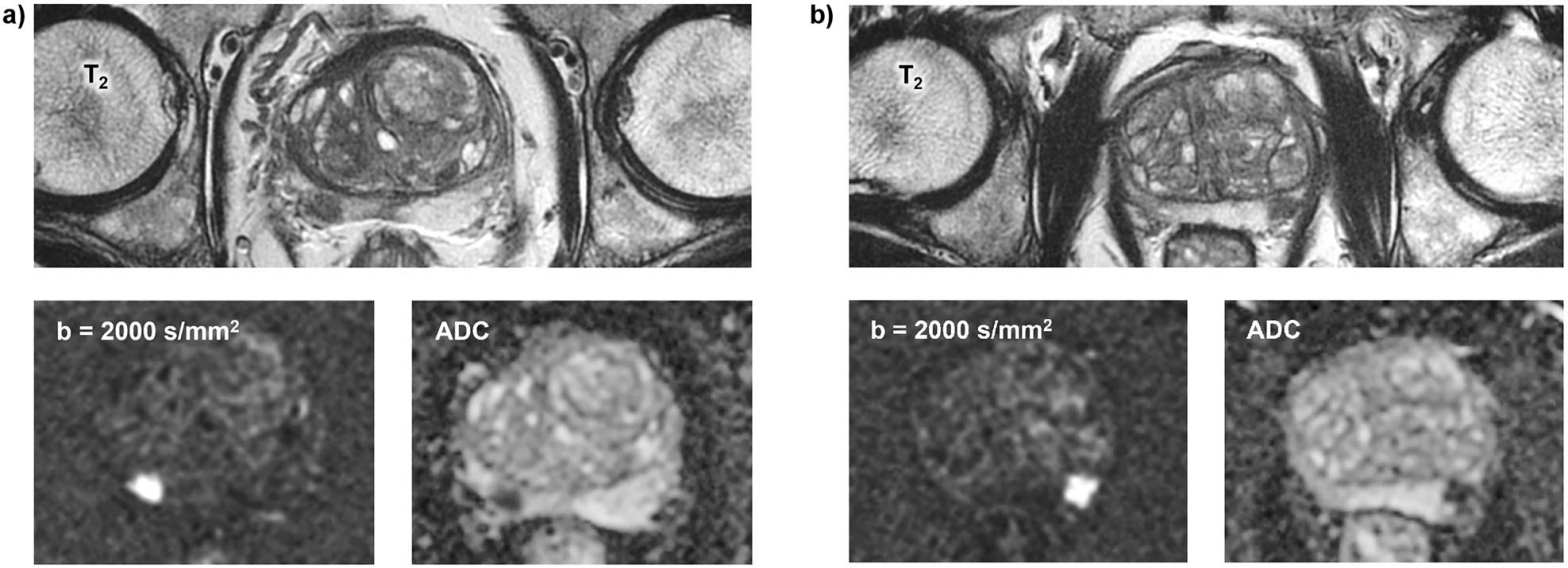
Example of patients where the visibility of the lesions is high due to a background score of A, as assessed by the expert reader. The T_2_-weighted image of each patient is complemented by the corresponding diffusion-weighted image and the corresponding ADC map: **a** Patient with a clearly visible lesion in the right part of the peripheral zone at the base. **b** Patient with a clearly visible lesion in the left part of the peripheral zone at the midgland

**Fig. 3:**
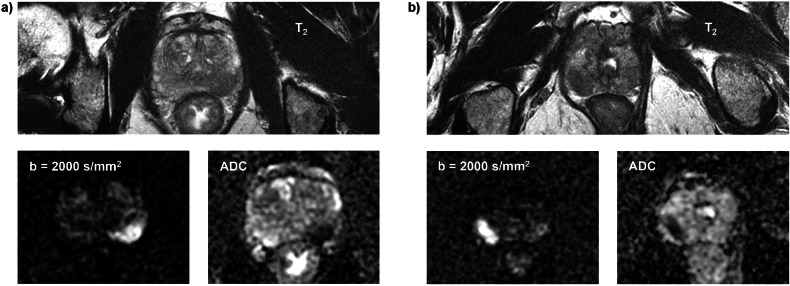
Example of patients where the visibility of the lesions is low due to a background score of B as assessed by the expert reader. The T_2_-weighted image of each patient is complemented by the corresponding diffusion-weighted image and the corresponding ADC map: **a** Patient with a lesion in the left part of the peripheral zone at the midgland. **b** Patient with a lesion in the left part of the peripheral zone at the apex

According to the expert reading, the patient cohorts with a background score of A and B consisted of 89 and 66 patients, respectively. The characteristics of the patient groups are listed in Table 1. Differences between groups regarding patient age (Cohen’s d = 0.05), PSA values (Cohen’s d = 0.2), and prostate volume (Cohen’s d = 0.4) were below the threshold (Cohen’s d < 0.5 for all parameters). In particular, there was no significant difference in the average age of patients with a background score of A (mean age = 66.5 years) and B (mean age = 66.2 years), respectively (delta = 0.3, Cohen’s d = 0.05, *p* = 0.77). Thus, there is little or no influence of these variables on the outcomes of interest, and they do not need to be considered when interpreting the results. Figures 2 and 3 show examples of patients in whom lesion visibility is high in patients with background levels of A and low in those with background levels of B, respectively.

**Table 1:** Characteristics of the patient groups

	Background score A	Background score B
Number of patients	89	66
Age (years)	66.5 (40–81)	66.2 (48–85)
Prostate volume (mL)	68.7 (21.0–180.0)	55.0 (17.0–252.0)
PSA values (ng/mL)	10.9 (1.2–45.0)	18.3 (0.7–440.0)
PSA density (ng/mL^2^)	0.16 (0.04–1.03)	0.33 (0.03–4.15)

Continuous data are reported as means and ranges

### Inter-reader agreement on background scores and on the presence of prostate cancer

Inter-reader agreement on background scores was substantial (Light’s kappa = 0.62, 95% CI = 0.54–0.71). There were 106 (68.4%) concordant readings. The pairwise comparisons between the readers are listed in Table 2.

**Table 2:** Pairwise Cohen’s kappa and 95% CI for the background score

	Reader 1	Reader 2	Reader 3
Reader 4	0.45 (0.32–0.58)	0.68 (0.56–0.79)	0.79 (0.69–0.89)
Reader 3	0.52 (0.39–0.65)	0.78 (0.68–0.88)	
Reader 2	0.52 (0.39–0.66)		

Agreement between the readers on the presence of prostate cancer was higher for a background score of A (Light’s kappa = 0.49, 95% CI = 0.38–0.61) than B (Light’s kappa = 0.34, 95% CI = 0.20–0.51) but did not reach statistical significance (difference = 0.15, t_126_ = 1.54, *p* = 0.063). The pairwise comparisons between the readers are listed in Table 3.

**Table 3:** Pairwise Cohen’s kappa and 95% CI on the presence of prostate cancer for the cohorts with a background score of A and B, respectively

Background score A			
	Reader 1	Reader 2	Reader 3
Reader 4	0.53 (0.36–0.70)	0.44 (0.26–0.62)	0.43 (0.25–0.60)
Reader 3	0.64 (0.48–0.81)	0.46 (0.27–0.65)	
Reader 2	0.45 (0.26–0.63)		

Background score B			
	Reader 1	Reader 2	Reader 3
Reader 4	0.31 (0.11–0.51)	0.42 (0.21–0.62)	0.45 (0.25–0.64)
Reader 3	0.33 (0.05–0.60)	0.07 (0.00–0.33)	
Reader 2	0.49 (0.23–0.75)		

### Sensitivity and specificity in the detection of cancer, depending on the background score

A total of 51 patients had to be secondarily excluded in this part of the analysis due to an indeterminate cancer status. The reasons were the following: (1) PI-RADS 2 and 3 exams with follow-up MRI showing progression (*n* = 6); (2) PI-RADS 3 exams without follow-up MRI (*n* = 11); (3) PI-RADS 4–5 exams with negative or no histological evidence (*n* = 8); (4) biopsy-proven Gleason 6 / ISUP grade group 1 without consecutive prostatectomy to assess cancer size and extraprostatic extension (*n* = 26). Of the remaining 104 patients, 52 had a clinically significant cancer, while 52 did not. Of the 52 patients without clinically significant cancer, 18 had biopsy-proven Gleason score < 6. Six patients had a follow-up MRI classified as PI-RADS ≤ 2, and 28 had an initial MRI classified as PI-RADS ≤ 2, with subsequent clinical and biological follow-up that did not require repeat MRI or biopsy. Details of the study population included in this analysis are provided in Table 4. Overall, a sensitivity of 86.61% (95% CI = 75.96–90.38%) and 89.42% (95% CI = 81.73–93.27%) was found for the cohorts with a background score of A and B, respectively. Specificity was 53.47% (95% CI = 44.44–61.11%) and 43.75% (95% CI = 29.69–54.69%) for the cohorts with a background score of A and B, respectively. Sensitivity and specificity for each reader and the results of the directional tests of two proportions are listed in Table 5. The results indicate that sensitivity is high regardless of the underlying background score, while specificity markedly decreased for a background score of B in readers with little experience, even if the effect is not significant.

**Table 4:** Characteristics of the patient groups included in the analysis of sensitivity and specificity in detecting clinically significant cancer in dependence of the background score

	Background score A	Background score B
Number of patients	62	42
Age (years)	66 (40–81)	67 (53–77)
Prostate volume (mL)	68.7 (21.0–180.0)	55.0 (17.0–252.0)
PSA values (ng/mL)	11.8 (4.0–45.0)	23.4 (0.7–440.0)
PSA density (ng/mL^2^)	0.17 (0.06–1.03)	0.43 (0.03–4.15)
Clinically significant cancer	26	26
Gleason score		
3 + 3	0	2
3 + 4	14	12
4 + 3	6	3
3 + 5	0	1
4 + 4	4	4
4 + 5	2	1
5 + 4	0	3

Continuous data are reported as means and ranges

**Table 5:** Sensitivity and specificity of each reader for the detection of clinically significant cancer in dependence of the background score, complemented by the results of the directional test of two proportions

	Sensitivity (%)	Statistics	Specificity (%)	Statistics
**Reader 1**				
Background score A	88.46 (65.38–96.15)	χ2_[1]_ = 0.27, *p* = 0.70	50.00 (30.56–63.89)	χ2_[1]_ = 1.90, *p* = 0.08
Background score B	96.15 (76.92–100.0)		25.00 (6.25–43.75)	
**Reader 2**				
Background score A	80.77 (57.69–88.46)	χ2_[1]_ < 0.01, *p* = 0.50	52.78 (33.33–66.67)	χ2_[1]_ = 1.29, *p* = 0.13
Background score B	84.62 (60.54–92.31)		31.25 (6.25–50.00)	
**Reader 3**				
Background score A	88.46 (65.38–96.15)	χ2_[1]_ = 0.27, *p* = 0.70	47.22 (27.78–61.11)	χ2_[1]_ < 0.01, *p* = 0.50
Background score B	96.15 (76.92–100.0)		50.00 (18.75–68.75)	
**Reader 4**				
Background score A	80.77 (57.69–88.46)	χ2_[1]_ = 0.00, *p* = 0.50	63.89 (44.44–75.00)	χ2_[1]_ < 0.01, *p* = 0.51
Background score B	80.77 (53.85–88.46)		68.75 (37.50–81.25)	

## Discussion

Routine assessment of the potential for diagnostic uncertainty may improve standardization and promote the appropriate integration of prostate MRI results into clinical decision-making. However, due to the constantly increasing workload, previously proposed background scores have not been integrated into routine clinical practice. The proposed easy-to-use binary background score, which is easier to integrate into daily routine, may close this gap while showing similar inter-reader agreement as previously proposed scores [9, 10]. A binary score is more tangible for radiologists than the previously proposed 4- and 5-point rating scales, which might increase clinical acceptance, especially given the variety of other scores used in radiology. Complemented with the recently proposed simplified version of the PI-QUAL score [5], the proposed background score may be used to communicate diagnostic certainty in routine clinical practice. A combination of both scores in prostate MRI reports is indicated, as they evaluate different factors that may influence diagnostic confidence in cancer detection, namely intrinsic factors reflecting physiological changes in the prostate on the one hand, and extrinsic factors reflecting technical image quality on the other. Combining PI-QUAL with the background score provides an operational framework for clinical use: PI-QUAL is first assessed to confirm adequate image quality, after which the background score is applied to identify cases in which normal signal heterogeneity may mimic or obscure cancer. This sequential approach can guide reader confidence, support double reading or expert review, and help standardize reporting, particularly for less experienced readers. However, it should also be mentioned that an earlier study observed a superior performance of the background score compared with the PI-QUAL score, i.e., a greater impact of the background score on the concordance of MRI and biopsy results [15]. The results of the present work indicate that inter-reader agreement on the presence of cancer is higher with a background score of A rather than B, reflecting greater diagnostic uncertainty for a background score of B. This assessment should be considered in the clinical decision-making process, although the results were not statistically significant. Based on our findings, we would have needed a total number of 384 patients to find a *p*-value below 0.05 at a power of 80%.

Sensitivity in detecting clinically significant cancer was high regardless of the value of the background score. This result is in contrast to previous studies in which a higher sensitivity was observed in patients with fewer changes in the normal background signal of the prostate [9, 10]. This discrepancy may be due to the higher b-value used in the present study, which may have increased tumor conspicuity on DWI to the extent that it compensated for the lower visibility of lesions on T_2_-weighted imaging in patients with a background score of B.

In addition to masking clinically significant prostate cancer, normal background signal changes can sometimes mimic tumors, leading to overdiagnosis and possible over-treatment [10, 16]. In the current study, in contrast to sensitivity, specificity decreased markedly in inexperienced readers for a background score of B rather than A. Reducing false-positive MRI results further remains a clinical priority, calling for risk-adapted strategies for patient biopsy selection. The current guidelines of the European Association of Urology are moving in this direction and already include a risk-adapted approach to the biopsy decision process that takes into account the MRI PI-RADS score and the PSA density [17]. A combination of PI-RADS, PSA density, PI-QUAL, and background score could enable a more refined risk stratification, providing both technical and physiological context, and thus help in the decision-making process for prostate biopsy.

We observed a difference in PSA and PSA density between patients with background score A and B. Patients with a background score B had a higher PSA and PSA density, which can be partly explained by the prostatitis responsible for the changes in background signal. Interpretation of PSA density by the urologist without considering the background score may lead to an overestimation of prostate cancer risk, also justifying the inclusion of the background score in prostate cancer risk stratification [18].

There are several limitations to our work. First, the present work was a retrospective study in a single center, and the results will have to be replicated in prospective cohorts at other institutions, vendors, and field strengths before clinical implementation of the score. Second, signal intensity on T_2_-weighted imaging in the peripheral zone varies with age [9], and lower physiological T_2_-weighted signal intensity is observed in younger screening populations. This could lead to younger patients being systematically assigned a background score of B more frequently than older patients, even if it was not the case in our patient population. This age-related effect could be comparable to that seen in breast imaging, where it is known that younger women typically have higher breast density and thus a more physiologically challenging background, which makes diagnostic interpretation more difficult [19]. For this reason, future studies should incorporate large patient groups that allow for stratifications across age groups so that the influence of age-related changes in T_2_-weighted signal intensity on the assignment of background scores can be evaluated. Third, in agreement with previous studies [9, 10, 15], the binary background score was defined on T_2_-weighted imaging, even though DWI is the primary determining sequence according to PI-RADS guidelines [2]. However, our clinical experience shows that T_2_-weighted imaging generally has good image quality in all centers, while DWI image quality is more dependent on patient preparation and the technical specifications of the DWI sequence used. In addition, previous work showed statistically significant positive correlation of T_2_-weighted imaging and DWI scores, but low inter-reader agreement for a background score determined on DWI [9]. Finally, signal heterogeneity on T_2_-weighted imaging has been shown to correlate positively with ADC heterogeneity and negatively with the average ADC in the peripheral zone [20], such that increasing signal heterogeneity on T_2_-weighted imaging (i.e., a background score of B instead of A) leads to lower tumor conspicuity on DWI. Fourth, to rule out insufficient technical image quality as a confounding factor, patients who were not assigned the maximum PI-QUAL score by one of the three readers were secondarily excluded. Future studies should evaluate whether technical image quality has an influence on the assignment of background scores. Fifth, in the analysis relative to histology, 51 patients had to be secondarily excluded, so that the total number of patients decreased to 104. This was done to ensure a high reference standard in the analysis, but larger patient populations are necessary to confirm our results.

In conclusion, the present work proposes an easy-to-use binary scoring system for the background signal intensity changes in the prostate that shows substantial inter-reader agreement. The background score allows radiologists and urologists to contextualize diagnostic confidence based on background visibility, with a background score B potentially indicating decreased specificity, particularly for readers with limited experience. Complemented by the recently modified PI-QUAL score, which assesses technical aspects of image quality, normal changes of the peripheral zone, which may interfere with diagnostic image interpretation, can be assessed in routine clinical practice.

## Supplementary information

ELECTRONIC SUPPLEMENTARY MATERIAL

## Data Availability

The data are not publicly available due to privacy or ethical restrictions.

## Abbreviations

CI: Confidence interval
DWI: Diffusion-weighted imaging
mpMRI: Multi-parametric MRI
PI-QUAL: Prostate imaging quality
PI-RADS: Prostate Imaging-Reporting and Data System
PSA: Prostate-specific antigen
