# Supplementary material for: Easy-to-use background score for routine prostate MRI
Source: Insights Imaging. 2026 Jan 26;17:23. doi: 10.1186/s13244-025-02200-5 (PMC12834886; doi:10.1186/s13244-025-02200-5)
Supplement: Supplementary file 1 — ELECTRONIC SUPPLEMENTARY MATERIAL [file 13244_2025_2200_MOESM1_ESM.pdf]

Easy-to-Use Background Score for Routine Prostate MRI

ELECTRONIC SUPPLEMENTARY MATERIAL

Supplemental Material 1, PI-QUAL scores stratified by background score for each reader

Reader	PI-QUAL score	Background score A	Background score B
Reader 1	maximum	133	37
	< maximum	14	16
Reader 2	maximum	122	70
	< maximum	3	5
Reader 3	maximum	94	67
	< maximum	24	15
